# Hyperbaric air mobilizes stem cells in humans; a new perspective on the hormetic dose curve

**DOI:** 10.3389/fneur.2023.1192793

**Published:** 2023-06-20

**Authors:** Kent J. MacLaughlin, Gregory P. Barton, Rudolf K. Braun, Julia E. MacLaughlin, Jacob J. Lamers, Matthew D. Marcou, Marlowe W. Eldridge

**Affiliations:** ^1^Department of Pediatrics, University of Wisconsin–Madison, Madison, WI, United States; ^2^Department of Internal Medicine, University of Texas Southwestern Medical Center, Dallas, TX, United States; ^3^Medical Oxygen Outpatient Clinic, The American Center, Madison, WI, United States

**Keywords:** CD34^+^, Traumatic Brain Injury, CD133, war veteran self harm, war veterans’ psychological suffering, war veterans suicide, hyperbaric air placebo, HBOT

## Abstract

**Introduction:**

Hyperbaric air (HBA) was first used pharmaceutically in 1662 to treat lung disease. Extensive use in Europe and North America followed throughout the 19th century to treat pulmonary and neurological disorders. HBA reached its zenith in the early 20th century when cyanotic, moribund “Spanish flu pandemic” patients turned normal color and regained consciousness within minutes after HBA treatment. Since that time the 78% Nitrogen fraction in HBA has been completely displaced by 100% oxygen to create the modern pharmaceutical hyperbaric oxygen therapy (HBOT), a powerful treatment that is FDA approved for multiple indications. Current belief purports oxygen as the active element mobilizing stem progenitor cells (SPCs) in HBOT, but hyperbaric air, which increases tensions of both oxygen and nitrogen, has been untested until now. In this study we test HBA for SPC mobilization, cytokine and chemokine expression, and complete blood count.

**Methods:**

Ten 34–35-year-old healthy volunteers were exposed to 1.27ATA (4 psig/965 mmHg) room air for 90 min, M-F, for 10 exposures over 2-weeks. Venous blood samples were taken: (1) prior to the first exposure (served as the control for each subject), (2) directly after the first exposure (to measure the acute effect), (3) immediately prior to the ninth exposure (to measure the chronic effect), and (4) 3 days after the completion of tenth/final exposure (to assess durability). SPCs were gated by blinded scientists using Flow Cytometry.

**Results:**

SPCs (CD45^dim^/CD34^+^/CD133^-^) were mobilized by nearly two-fold following 9 exposures (*p* = 0.02) increasing to three-fold 72-h post completion of the final (10th) exposure (*p* = 0.008) confirming durability.

**Discussion:**

This research demonstrates that SPCs are mobilized, and cytokines are modulated by hyperbaric air. HBA likely is a therapeutic treatment. Previously published research using HBA placebos should be re-evaluated to reflect a dose treatment finding rather than finding a placebo effect. Our findings of SPC mobilization by HBA support further investigation into hyperbaric air as a pharmaceutical/therapy.

## Introduction

Presently, hyperbaric air (HBA) is not thought of as a medication. Its singular approved medicinal use is for Acute Mountain Sickness. Historically it has been used medicinally, initially reported by Henshaw in 1662 to treat “afflictions of the lungs” (1) and additional reports from Europe and America followed (1). In 1857 Simpson published a paper using HBA to treat lung pathologies including tuberculosis (2). Interest in hyperbaric air as a medication surged following successful treatments of “Spanish flu” patients by Cunningham in 1918 (3–5). Unfortunately, Cunningham produced a paucity of papers supporting his work and when he died in 1937, interest in hyperbaric air abated.

HBA began to be employed again in the modern era with controversial results. It was used as a placebo by Collet while investigating the use of hyperbaric oxygen in Cerebral Palsy (6) and also as a placebo in brain injury by Miller, Wolf and Cifu (7–9). In each of these experiments, both the treatment group and the placebo group improved revealing an apparent placebo effect. The use of hyperbaric air as a placebo is energetically debated and controversial because it increases the oxygen tension. In contrast to the placebo findings, similar published studies not using the hyperbaric air placebo, found significant improvements in brain injury (10–20).

Considering the aforementioned, we asked the question, “is hyperbaric air an appropriate placebo?”. We searched the literature and found no evidence that hyperbaric air has been tested. We designed a test of hyperbaric air using a gold standard endpoint of oxygen therapy, stem cell mobilization.

Our specific aims were (1) to characterize stem cell mobilization in healthy adults following daily exposures to hyperbaric air, (2) determine if other biomarkers were modulated, (3) determine if there were acute changes, and (4) if so, were the changes durable. Based on previous research done in our lab (21) and calculating a 27% increase in oxygen partial pressures in the inhaled gases, we hypothesized that (1) stem cells would be mobilized, (2) biomarkers would be modulated (3) there would be acute changes and (4) the stem cell mobilization would be durable.

## Materials and methods

### Design and subjects

This prospective hyperbaric air study was a randomized, single-blind study conducted at the University of Wisconsin – Madison Clinical Sciences Center between May 1st, 2021, and August 31st, 2021. This study is approved by the Institutional Review Board of the University of Wisconsin – Madison. UW IRB ID: 2020-0293-CR001. All participants provided written informed consent. Healthy adults were recruited for participation in this study (Table 1).

**Table 1:** Anthropometric data of subjects in this hyperbaric air study.

	*N*	Mean	Standard deviation
Age	10	34.54 years	1.36 years
Height	10	171.75 cm	9.38 cm
Weight	10	81.40 kg	21.05 kg
BMI	10	27.43%	2.8%
Female	5	5	NA

### Hyperbaric air exposure

All exposures were at 1.27 ATA (4 psig) of room air for 90 min in a Gamow style Mountain Sickness Chamber (Hyperbaric Technologies Inc. Amsterdam, NY, United States). To minimize circadian cell cycle variations including acrophase and circannual cycle, all subjects were diurnally active and all hyperbaric exposures and sample collections occurred at the same time of day over the contiguous 15-day experiment.

### Sample collection

**Figure 1:**
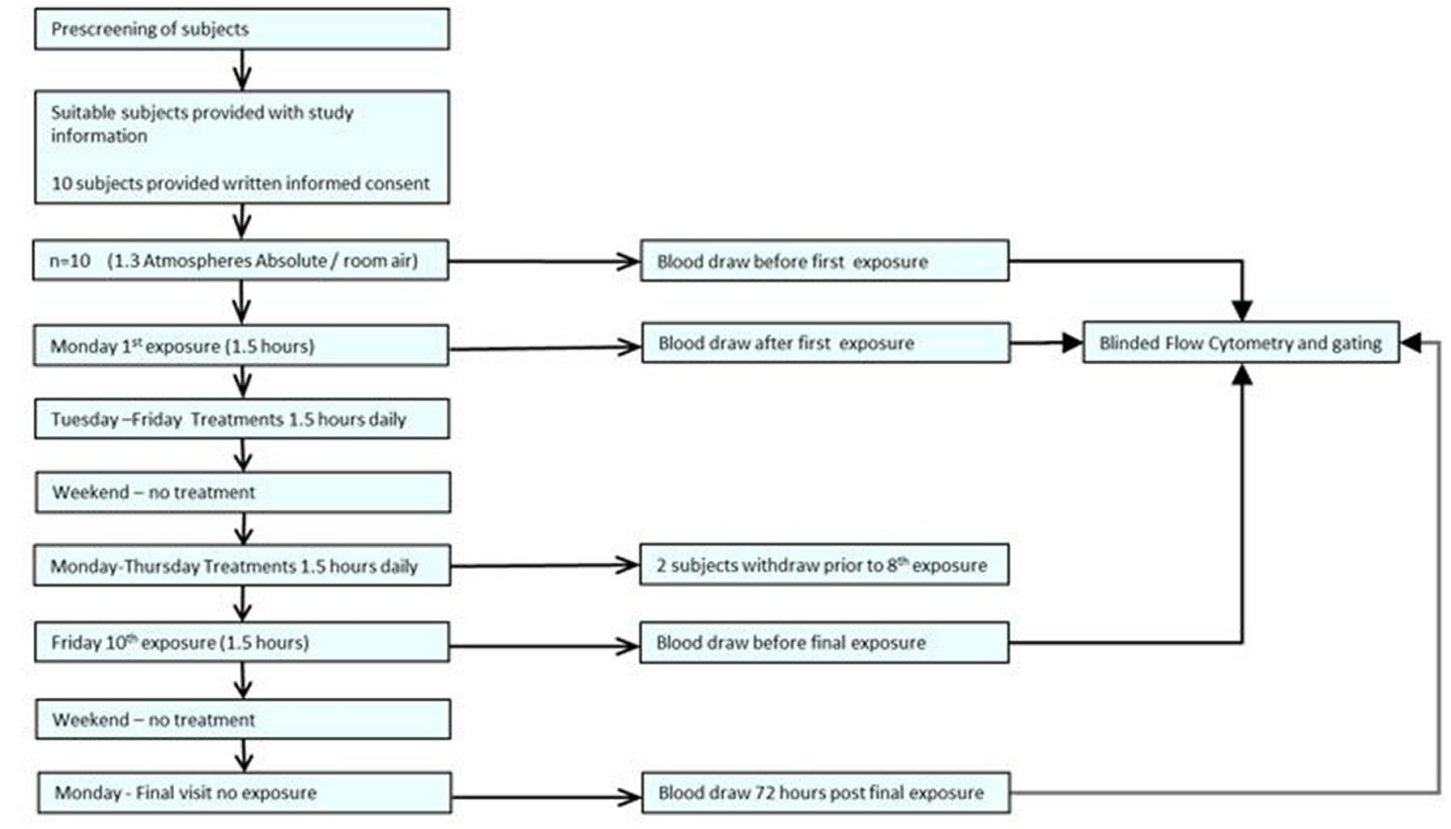
Experimental protocol.

Each peripheral venous blood sample was collected during the subject’s exposure time slot using either a 21- or 23-gauge BD Vacutainer Safety-Lok Blood Collection Set (Becton, Dickinson and Company, Franklin Lakes, NJ, United States) into a Cyto-Chex BCT tube (Streck Inc. NE USA), BD Vacutainer Plastic Blood Collection Tubes with K2EDTA, BD Vacutainer Plastic Blood Collection Tubes – PST Plasma Separation Tubes, and Greiner Bio-One K2EDTA GelTubes. All samples were stored according to manufacturer’s directions. Study protocol in graphic format is included in Figure 1.

### Flow cytometry

Fluorescence minus one (FMO) tubes, rainbow bead tubes, and single antibody tubes were prepared as gating references. Antibodies were pipetted into flow cytometry tubes according to manufacturer’s instructions. Antibodies used include CD34 = Brilliant Violet 421 (BioLegend, San Diego, CA, United States), CD45 = Alexa Fluor 488 (BioLegend, San Diego, CA, United States), CD133 = PE (Miltenyi Biotec, North Rhine-Westphalia, Germany) CD31 = Brilliant Violet 605 (BioLegend, San Diego, CA, United States), CD105 = PE-Cy7 (BioLegend, San Diego, CA, United States), Ghost Dye Red 780 = Tonbo Biosciences, San Diego, CA.

Flow cytometry was performed by blinded scientists on a ThermoFisher Attune NxT (Waltham, MA, United States). Samples were analyzed by blinded scientists using FlowJo software (FlowJo, Ashland, OR, United States).

### Enzyme-linked immunosorbent assay

The Invitrogen ProcartaPlex^™^ Human Immune Monitoring Panel 65-Plex (Invitrogen, Waltham, MA, United States) was used to assess changes in cytokines, chemokines and growth factors. All tests were performed by blinded scientists at the University of Wisconsin Non-Human Primate Research Center (Madison, WI, United States).

### Statistical analysis

The first blood draw taken prior to the first exposure served as the control. To determine whether there was an overall effect across exposures, we utilized the Friedman test (nonparametric alternative to one-way ANVOA with repeated measures) and if significant, comparisons between all-time points were performed using the Wilcoxon signed rank test. Significance level was determined *a priori* at the 0.05 level and all tests were two-tailed. Statistical analyses were calculated using Graph Pad Prism (GraphPad Prism 9.0.0 Software, San Diego, CA, United States).

## Results

### CD45^dim^

#### Increased frequency of CD45^dim^/CD34^+^/CD133^−^ following nominal exposure to hyperbaric room air

**Figure 2:**
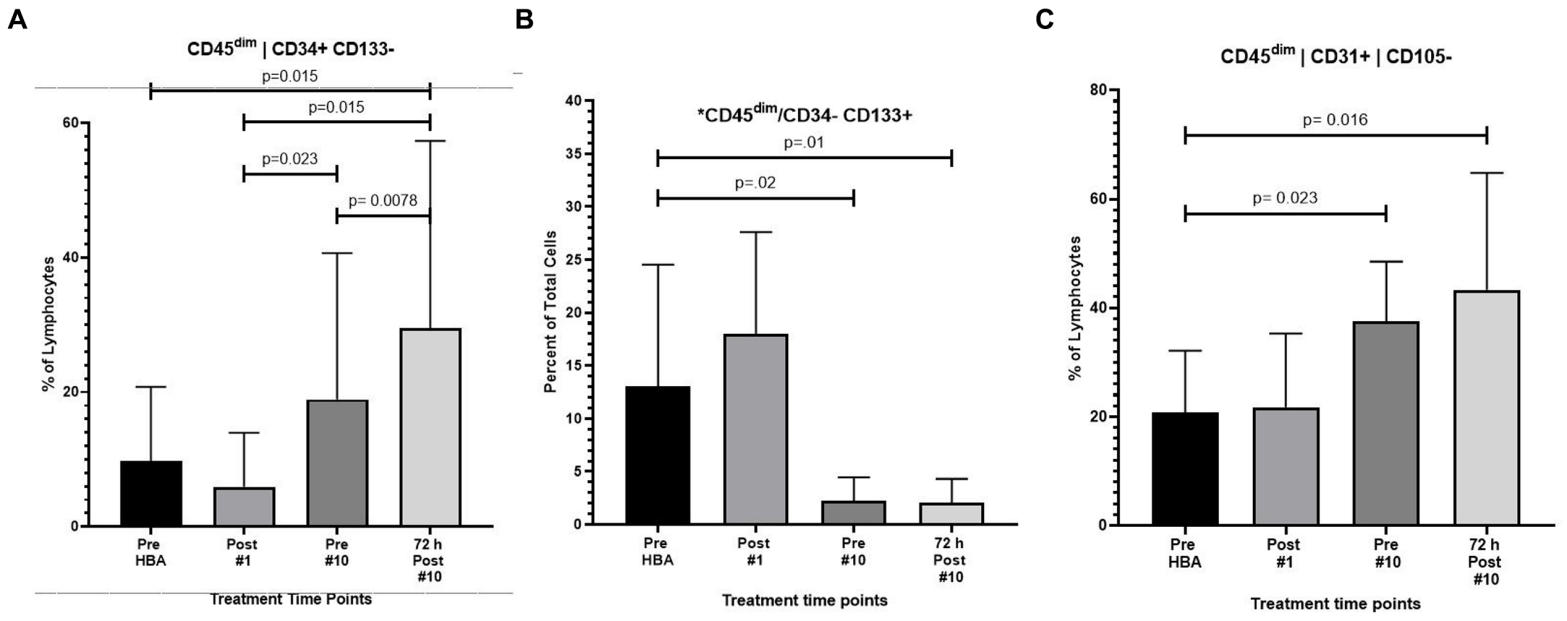
Frequency of CD45^dim^/CD34^+^/CD133^-^, CD45^dim^/CD34^-^/CD133^+^ and CD45^dim^/CD31^+^/CD105^-^after intermittent hyperbaric air exposure detected by flow cytometry. **(A)** CD34^+^ and CD133^-^ stem progenitor cells. **(B)** CD34^-^ and CD133^+^ stem progenitor cells. **(C)** CD31^+^ and CD105^-^ stem progenitor cells.

As previously described 10 humans were exposed to 1.27 ATA of room air 10 times over the course of a 12-day period. Results revealed a significant increase in the frequency of CD45^dim^/CD34^+^/CD133^−^ stem progenitor cells in venous blood resulting in an approximate two-fold increase directly prior to the 10th exposure (*p* = 0.02). CD45^dim^/CD34^+^/CD133^−^ SPCs continued to increase during the 3 days following the end of exposures and increased to three-fold, 72 h after the 10th exposure (*p* = 0.008); (Figure 2A).

#### Decreased frequency of CD45^dim^/CD34^−^/CD133^+^ after hyperbaric exposure

While CD45^dim^/CD34^+^/CD133^−^ SPCs increased, the frequency of CD45^dim^/CD34^−^/CD133^+^ primitive pro-angiogenic stem progenitor cells significantly decreased after exposure to intermittent hyperbaric air. CD45^dim^/CD34^−^/CD133^+^ decreased by nearly five-fold prior to the 10th exposure (*p* = 0.02) and mobilization decreased by six-fold 72 h after the 10th exposure (*p* = 0.01) (Figure 2B).

#### Increased frequency of CD45^dim^/CD31^+^/CD105^−^ after hyperbaric exposure

The frequency of CD45dim/CD31^+^/CD105^-^ was also significantly increased after hyperbaric exposure by nearly two-fold prior to the 10th exposure (*p* = 0.023) and increased to over two-fold 3 days after the 10th exposure (*p* = 0.016) (Figure 2C).

### Mobilization of CD45^+^/CD34^+^/CD133^−^

**Figure 3:**
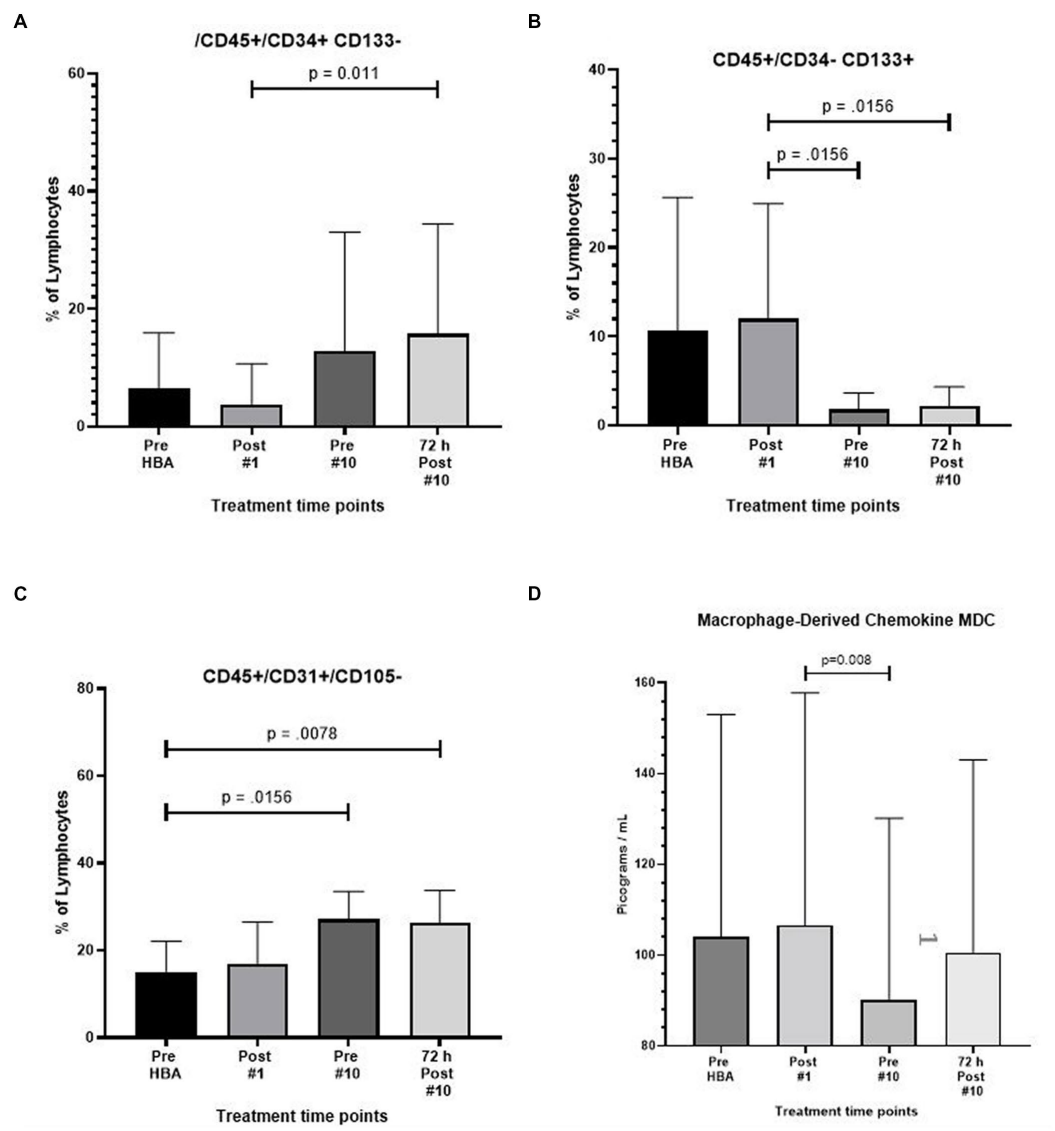
Frequency of CD45^+^/CD34^+^/CD133^-^, CD45^+^/CD34^-^/CD133^+^, CD45^+^/CD31^+^/CD105^-^, and macrophage derived chemokine after intermittent hyperbaric air exposure detected by flow cytometry and ELISA. **(A)** CD34^+^ and CD133^-^ stem progenitor cells. **(B)** CD34^-^ and CD133^+^ stem progenitor cells. **(C)** CD31^+^ and CD105^-^ stem progenitor cells. **(D)** Macrophage derived chemokine.

The expression of CD45^+^/CD34^+^/CD133^-^ also increased by nearly 3.5 fold after 9 full exposures (*p* = 0.012) and increased to over four fold 72 h following the end of the 10th exposure (*p* = 0.002) (Figure 3A).

### Decreased frequency of CD45^+^/CD34^−^/CD133^+^

A significant reduction in the expression of CD45^+^/CD34^−^/CD133^+^ was noted resulting in a six-fold reduction prior to the 10th exposure (*p* = 0.016) and recovering to 5.4-fold 72 h after the 10th and final exposure (*p* = 0.016) (Figure 3B).

### Mobilization of CD45^+^/CD31^+^/CD105^−^

The expression of CD45^+^/CD31^+^/105^-^ increased by nearly two-fold after 9 full exposures (*p* = 0.02) and remained at the level through 72 h following the end of the 10th exposure (*p* = 0.008) (Figure 3C).

### Complete results from flow cytometry

Complete results from flow cytometry testing, including non-significant findings are included in tabular form for reference. (Table 2).

### Macrophage-derived chemokine (MDC) expression significantly decreased

Results from the Invitrogen ProcartaPlex^™^ Human Immune Monitoring Panel 65-Plex ELISA like analysis showed there was only one change to report. The expression of Macrophage-derived Chemokine (MDC) was significantly lower between the second and third time points (*p* = 0.008). All other tests revealed no change (Figure 3D).

### Complete blood count with differential

There were no significant changes in CBC at any time points (Table 2).

**Table 2:** Summary of cell type frequency after initial and intermittent exposure detected by flow cytometry.

	CD45^dim^	CD45^+^	CD45^−^
CD45^dim^	=,=,=,=		
CD45^+^		=,=,=,=	
CD45^_^			=,=,↓,=
CD31^_^ CD105^+^	=,=,=,=	=,=,=,=	=,=,=,=
CD31^+^ CD105^_^	=,=,↑,↑	=,=,↓,↓	=,=,=,=
CD31^+^ CD105^+^	=,=,=,=	=,=,=,=	=,=,=,=
CD31^_^ CD105^_^	=,=,=,=	=,=,=,=	=,=,=,=
CD34^_^ CD133^+^	=,=,↓,↓	=,=,↓,↓	=,=,=,=
CD34^+^ CD133^_^	=,=,↑,↑	=,=,↑,↑	=,=,=,=
CD34^+^ CD133^+^	=,=,=,=	=,=,=,=	=,=,=,=
CD34^_^ CD133^_^	=,=,=,=	=,=,=,=	=,=,=,=

Results from four time points represented by change symbol separated by commas. The = symbol represents no change in frequency, the ↓ symbol represents a significant reduction in frequency, and the ↑ symbol indicates a significant mobilization. The first time point is the control point taken prior to exposure. The second is taken directly after the first exposure. The third timepoint was taken just prior to the 10th and final exposure and the fourth time point is the frequency 3 days after cessation of exposures.

## Discussion

The smallest dose of hyperbaric air that will result in a therapeutic effect is unknown and strongly debated among scientists and physicians. Pressures below 1.4 atmospheres absolute of hyperbaric air are accepted as a placebo but have not previously been tested in humans. Studies using hyperbaric air as a placebo have resulted in a “placebo” or “participation” effect (7–9). This placebo effect finding is vigorously disputed because hyperbaric air significantly increases the partial pressure of oxygen (and nitrogen) in the inspired air (22–27). The “placebo effect” findings have effectively restricted the use of HBOT for many conditions including Traumatic Brain Injury in soldiers returning from combat.

In this study we asked the question, will a small dose of hyperbaric air (1.27 ATA), that is below the accepted 1.4 ATA therapeutic threshold, mobilize stem cells similar to HBOT? We hypothesized that stem cells would be mobilized.

Indeed, stem cells were significantly mobilized, refuting previous “placebo effect” findings. We intend that the results will provide needed experimental data to medical societies and journal editors, and in turn provide guidance to FDA and physicians.

### Critical analysis of major findings

Testing for stem cell mobilization using flow cytometry and gated by blinded scientists, the major finding of this study is that 1.27 ATA hyperbaric air mobilizes CD34^+^/CD133^-^ SPCs and CD31^+^/CD105^-^ stem cells in humans receiving 10 daily 90-min exposures (Figures 2A,C, respectively). Mobilization of CD34^+^ SPCs has also been observed in isobaric hyperoxic (21) and in hyperbaric hyperoxic conditions (28, 29).

The mechanism of hyperbaric air SPC mobilization in this experiment is beyond the scope of this study, but may be similar to SPC mobilization found in hyperbaric oxygen therapy, which activates nitric oxide synthase and plays a prime role in initiating CD34^+^ SPC mobilization (30–33). (CD34 background) CD34^+^ adult stem/progenitor cells are a group of specific cell types that possess the abilities of self-renewal and multipotent differentiation (34, 35).

CD34^+^ is expressed on hematopoietic and pro-angiogenic stem progenitor cells and on endothelium (36). Pro-angiogenic stem/progenitor cells contribute to neovascularization by a process of homing to ischemic tissue called vasculogenesis, and by budding endothelium from established blood vessels in a process called angiogenesis (37, 38). Further research is needed to understand the homing and function of the CD34^+^ SPCs mobilized by intermittent hyperbaric air exposures.

A second significant finding of this study, which we report for the first time, is hyperbaric air mobilizes CD31^+^/CD105^-^ SPCs. This novel finding has many implications in the field of hyperbaric air and hyperbaric oxygen. (CD31^+^ background) CD31 is thought to have a protective role in experimental atherosclerosis (39). The therapeutic potential of CD31 agonists to manage atherosclerotic disease manifestations is a consistent finding in pre-clinical studies (40). Although this is the first time that CD31 has been reported to be mobilized by either hyperbaric air or hyperbaric oxygen, it is not unexpected, given that CD31 is associated with endothelial function much like CD34 and CD133. It was beyond the scope of this research project to determine if these mobilized CD31 stem cells participate in wound healing and angiogenesis. Future research testing the effect of hyperbaric air mobilized CD31 stem cells on healing, angiogenesis, and atherosclerotic disease states would be prudent.

Our results also revealed an interesting relationship between SPCs expressing CD45^dim^/CD34^+^/CD133^−^ and those SPCs expressing CD45^dim^/CD34^−^/CD133^+^. CD133+ SPCs are hematopoietic precursors to CD34^+^ and almost all hematopoietic pluripotent and committed stem cells in colony-forming assays express CD34^+^ (36). In this study we found that CD34^+^ SPCs increased while at the same time CD133^+^ SPCs decreased. We hypothesize that the exposure to intermittent hyperbaric air may mobilize CD133^+^ from bone marrow, and also play a role in the differentiation of the CD133^+^ primitive hematopoietic precursor into the CD34^+^ SPC. However, it has also been shown that CD133^+^ have a high capacity to differentiate into other cell types including fibroblasts, hepatocytes, and neural cell-types (35). Unfolding the explanation for the significant reduction in the expression of CD45^dim^/CD34^−^/CD133^+^ expressing SPCs holds exciting potential.

Previous research has shown that breathing 100% oxygen at 2.4 atmospheres absolute mobilized stem/progenitor cells at a significantly greater rate than 2.0 Atmospheres Absolute suggesting a dose effect (41). HBOT at 2.0 ATA (P_I_O^2^ of 1426 mmHg and P_I_N^2^ of 0 mmHg) resulted in a two-fold mobilization of CD34^+^ SPCs after 1 exposure and an eight-fold increase after 20 exposures (28). In this study using 1.27 ATA of hyperbaric air (P_I_O^2^ of 189 mmHg and P_I_N^2^ of 706 mmHg) we saw a two-fold increase in SPC mobilization just prior to the 9th exposure. This supports the hypothetical relationship between oxygen dose and stem cell mobilization.

Another aim of this study was to determine if the stem progenitor cell mobilization was durable. Because the stem progenitor cell mobilization increased another fold following the end of exposures a durable effect is likely.

Interestingly, we also found changes in cells expressing CD45^+^ cell subtypes. These changes were remarkably similar to changes in the CD45dim population. CD45^+^ is expressed on all hematopoietic cells, including HSCs and osteoclasts, which are of hematopoietic origin (42), and is known as a pan-leukocyte marker.

Macrophage-derived chemokine (MDC) expressed in venous blood significantly decreased prior to the ninth exposure and returned to pre-exposure levels 72 h following the final exposure. The expression of MDC is increased in idiopathic pulmonary fibrosis (43) and elevated following resuscitation of patients after hemorrhage. Macrophage-derived chemokine may provide a therapeutic strategy to mitigate this inflammatory response (44). MDC is also thought to serve as a marker of pharmacological therapy response in Major Depressive Disorder (45).

Changes in barometric and hydrostatic pressure may be a mechanism of hyperbaric therapy. Small changes in atmospheric pressure elicit responses in many organisms (46). However the mechanism(s) is poorly understood. One possibility is that pressure is sensed through hydrostatic compression of heterogeneous structures. For instance, cells in suspension, including platelets (47, 48) and cartilage cells (49, 50), respond to small changes in pressure ostensibly through this mechanism. Another possibility is that cellular structures may produce shear and strain through differential compression. Microtubules, actin and other cytoskeletal proteins respond to this type of local mechanical stresses (51, 52). It should also be noted that certain animals appear to respond to changes in atmospheric (air) pressure. For example, pigeons exhibit changes in heart rate in response to changes of about 1 mbar, which is a typical daily fluctuation in barometric pressure. But in such cases, it is unclear how barometric pressure changes are sensed (53). It is probable that changes in pressure have effects on human physiology.

Another possible mechanism ultimately resulting in stem cell mobilization in this experiment is a progressive accumulation of endogenous anti-oxidants at the cellular level. These antioxidants accrue in response to repeated exposure to a hyperoxic environment. The result is an up-regulation of the Hypoxia-inducible factor 1α (HIF-1α) transcription factor activity (54–60). This hypothetical mechanism is best described as the Normoxic Hypoxic Paradox. This mechanism is best characterized by an increase in reactive oxygen species (ROS) due to the hyperoxic cellular environment. The increased cellular ROS creates an imbalance of ROS/scavenger antioxidants ratio. The increased ROS molecules initially hydroxylate most of the HIF-1α, facilitating ubiquitination and degradation of most of the HIF-1α subunits. However, it is postulated that an adaptive response to repeated hyperoxia, increases the production of scavengers in proportion to the increased ROS generation. The ROS/scavenger ratio gradually becomes balanced in the hyperoxic environment. However, when the hyperoxic exposure ends and the cell returns to a normoxic state, the ROS/ scavenger ratio is again imbalanced, but this time with more scavengers than ROS, which produces a reduced ROS environment. Less ROS leads to an increase in HIF-1α, initiating HIF transcription factor activity, resembling a hypoxic state, but in a normoxic environment. In this study CD34^+^/CD133^-^ expression trended downward directly following the initial hyperbaric air exposure, but increased significantly prior to the tenth exposure. The opposite was true with CD34^-^/CD133^+^. These results support the hypothetical Normoxic Hypoxic Paradox mechanism.

While our study clearly showed that the hyperbaric air dose that was used as a placebo mobilizes proangiogenic and hematopoietic stem progenitor cells and likely has a therapeutic physiologic effect similar to hyperbaric oxygen therapy (29, 61–63), it was beyond the scope of this study to determine clinical significance. Hopefully our results will generate renewed interest in hyperbaric air and future studies will investigate both mechanism and clinical outcomes.

### Limitations

The major limitation of our study is a relatively small sample size.

### Future directions

It should not be overlooked that although we increased the oxygen partial pressure and presume that oxygen is the active element in hematopoietic and pro-angiogenic stem progenitor mobilization, the partial pressure of nitrogen and remaining trace gasses are also increased. Nitrogen is not an inert element and can form five potential oxidation states and three potential reduction states with various levels of reactivity (64). Recent evidence suggests that intracellular reactive oxygen and nitrogen species play an important role in intracellular signaling cascades (65). However, little is known about the effect of nitrogen and trace gasses on SPC mobilization or cytokine, chemokine and growth factor modulation. Increased nitrogen and trace gasses as a stimulator of SPC mobilization and chemokine modulation is a possible mechanism in this research project and an exciting idea to explore in future research.

It is very possible that stem cell mobilization by hyperbaric air provides an additional modality to heal injuries. Because of its reduced cost of delivery, it may prove beneficial in developing nations and in underserved populations. Hyperbaric air also increases safety because the oxygen partial pressure is relatively low. The low oxygen partial pressure in hyperbaric air may also provide increased therapeutic value by not overloading the cerebral energy metabolism balance (66, 67).

Finally, our data suggests that the therapeutic dose of oxygen begins at a much smaller partial pressure than previously thought and adds a new data point on the initial portion of the hormetic curve of oxygen dose.

Although we found that intermittent small increases in hyperbaric air pressure mobilized stem cells, this study should not be taken as an endorsement of the use of intermittent hyperbaric air for any purpose other than the indications approved by the FDA.

Why are these findings important? First, prior to this research it was not known that breathing a small increase in hyperbaric air would mobilize stem progenitor cells. This knowledge could be an important low-cost healthcare option when hyperbaric oxygen is not available, especially in underserved populations, remote areas, and developing nations. Second, because the technology is lightweight and portable, it is hypothetically possible to be used when transporting combatants and civilians out of a warzone to extend the viability of damaged tissue and to reduce exacerbating gas embolism when air-lifting by high altitude flights is required. Finally, this research refutes the findings of a placebo effect in the decades-old use of slightly pressurized room air as a placebo in hyperbaric oxygen research.

## Conclusion and impact

In this study we demonstrate for the first time that intermittent exposure to ostensibly insignificant pressures of hyperbaric air mobilizes stem progenitor cells in a similar manner to that seen in isobaric hyperoxia and hyperbaric oxygen therapy (21, 28, 29, 41). We also establish that the stem progenitor cell mobilization is durable.

This research reveals that hyperbaric air, even at an ostensibly insignificant dose, has significant effects on human physiology, is not a placebo, and should be considered as an active physiologic intervention. Its use as a pharmaceutical should be investigated further.

Although this study did not test for clinical results, clinical outcomes of hyperbaric air have been reported in similar hyperbaric air studies (6–8). This study supports the data, but refutes the conclusions in those studies by revealing a mechanism of action for the clinical improvements reported in the hyperbaric air group in those studies. Much more work is needed to develop protocols of hyperbaric air dose that provide the maximum therapeutic benefit.

These findings substantiate the need for testing hyperbaric air doses prior to using hyperbaric air as a placebo in scientific investigations. These findings also substantiate the urgent need for reevaluation of findings in historical studies using hyperbaric air placebos. Our findings suggest that these historical placebo-controlled studies were not placebo-controlled studies. Paradoxically, our findings indicate that they were dose studies and the findings of a “placebo effect” or “participation effect” are inherently flawed. The “findings” and “conclusions” in studies using hyperbaric air as a placebo should be reevaluated from a dose study perspective. Finally, we hope the findings in this study will persuade the medical societies around the world to consider reevaluation of their definition of hyperbaric medicine to include nominal hyperbaric air.

Looking back at the “Hyperbaric Air” work of Henshaw, Simpson and Cunningham, our findings support their reports.

## Data availability statement

The original contributions presented in the study are included in the article/supplementary material, further inquiries can be directed to the corresponding author.

## Ethics statement

The studies involving human participants were reviewed and approved by the University of Wisconsin–Madison. The patients/participants provided their written informed consent to participate in this study.

## Author contributions

KM and GB: ideas conception. KM, GB, RB, and ME: study and experiments design. KM, JM, RB, JL, and MM: experiments perform, data acquisition, and analysis. GB and RB: guidance and critical feedback on data acquisition and analysis. RB and ME: project supervision. KM: manuscript writing. All authors provided critical feedback and contributed to the final version of manuscript.

## Funding

This work was supported by the Foundation for the Study of Inflammatory Disease and the International Hyperbaric Association.

## Conflict of interest

The authors declare that the research was conducted in the absence of any commercial or financial relationships that could be construed as a potential conflict of interest.

## Publisher’s note

All claims expressed in this article are solely those of the authors and do not necessarily represent those of their affiliated organizations, or those of the publisher, the editors and the reviewers. Any product that may be evaluated in this article, or claim that may be made by its manufacturer, is not guaranteed or endorsed by the publisher.
